# Evidence that duplications of 22q11.2 protect against schizophrenia

**DOI:** 10.1038/mp.2013.156

**Published:** 2013-11-12

**Authors:** E Rees, G Kirov, A Sanders, J T R Walters, K D Chambert, J Shi, J Szatkiewicz, C O'Dushlaine, A L Richards, E K Green, I Jones, G Davies, S E Legge, J L Moran, C Pato, M Pato, G Genovese, D Levinson, J Duan, W Moy, H H H Göring, D Morris, P Cormican, K S Kendler, F A O'Neill, B Riley, M Gill, A Corvin, Wellcome Trust Case Control Consortium, N Craddock, P Sklar, C Hultman, P F Sullivan, P V Gejman, S A McCarroll, M C O'Donovan, M J Owen

**Affiliations:** 1 MRC Centre for Neuropsychiatric Genetics and Genomics, Institute of Psychological Medicine and Clinical Neurosciences, Cardiff University, Cardiff, UK; 2 Department of Psychiatry and Behavioral Sciences, NorthShore University HealthSystem, Evanston, IL, USA; 3 Department of Psychiatry and Behavioral Sciences, University of Chicago, Chicago, IL, USA; 4 Stanley Center for Psychiatric Research, Broad Institute of MIT and Harvard, 7 Cambridge Center, Cambridge, MA, USA; 5 Biostatistics Branch, Division of Cancer Epidemiology and Genetics, National Cancer Institute, Bethesda, MD, USA; 6 Department of Genetics, University of North Carolina, Chapel Hill, NC, USA; 7 School of Biomedical and Biological Sciences, Plymouth University, Plymouth, UK; 8 Department of Psychiatry and Behavioral Science, Zilkha Neurogenetic Institute, University of Southern California, Los Angeles, CA, USA; 9 Department of Psychiatry and Behavioral Sciences, Stanford University, Stanford, CA, USA; 10 Department of Genetics, Texas Biomedical Research Institute, San Antonio, TX, USA; 11 Department of Psychiatry and Neuropsychiatric Genetics Research Group, Institute of Molecular Medicine, Trinity College Dublin, Dublin 2, Ireland; 12 Department of Psychiatry and Human Genetics, Virginia Institute of Psychiatric and Behavioral Genetics, Virginia Commonwealth University, Richmond, VA, USA; 13 Department of Psychiatry, Queen's University, Belfast, Northern Ireland; 14 Division of Psychiatric Genomics, Department of Psychiatry, Icahn School of Medicine at Mount Sinai, New York, NY, USA; 15 Department of Medical Epidemiology and Biostatistics, Karolinska Institutet, Karolinska, Sweden; 16 Department of Genetics, University of North Carolina at Chapel Hill, Chapel Hill, NC, USA; 17 Department of Medical Epidemiology and Biostatistics, Karolinska Institutet, Stockholm, Sweden; 18 Department of Psychiatry, University of North Carolina at Chapel Hill, Chapel Hill, NC, USA

**Keywords:** 22q11.2, CNV, duplication, protective, schizophrenia

## Abstract

A number of large, rare copy number variants (CNVs) are deleterious for neurodevelopmental disorders, but large, rare, protective CNVs have not been reported for such phenotypes. Here we show in a CNV analysis of 47 005 individuals, the largest CNV analysis of schizophrenia to date, that large duplications (1.5–3.0 Mb) at 22q11.2—the reciprocal of the well-known, risk-inducing deletion of this locus—are substantially less common in schizophrenia cases than in the general population (0.014% vs 0.085%, OR=0.17, *P*=0.00086). 22q11.2 duplications represent the first putative protective mutation for schizophrenia.

Large, rare copy number variants (CNVs) at several genomic loci increase risk for schizophrenia and other neurodevelopmental disorders including intellectual disability (ID), autism spectrum disorders and attention-deficit hyperactivity disorder.^1, 2^ The first CNV to be unequivocally implicated in schizophrenia was the 22q11.2 deletion, which also causes most cases of DiGeorge and Velocardiofacial Syndromes (OMIM #188400 and #192430).^3^ The 22q11.2 deletion is estimated to occur in about 1 in 4000 live births^4^, and is one of the most common CNVs associated with ID.^1^ It is also the strongest known specific risk factor for developing schizophrenia in adulthood.^5, 6^ 22q11.2del is also associated with psychiatric problems in childhood such as attention-deficit hyperactivity disorder, autism, depression and anxiety as well as a range of physical phenotypes.^7^

CNVs arise at this locus from non-allelic homologous recombination between low copy repeats^8^ and, as a result, the deletions occur in a set of low copy repeat-dependent sizes, the majority being about 3 Mb, and most of the remainder (<10%) being nested deletions of 1.5 Mb within that region.^9^ Even among those with the same sized CNV, the phenotype of 22q11.2del carriers is highly heterogeneous with respect to physical, psychiatric and cognitive sequelae,^9^ suggesting the involvement of other genetic, environmental or stochastic factors.

Given the non-allelic homologous recombination mechanism by which deletions are produced, it is not surprising that reciprocal 22q11.2 duplications also arise in human populations,^10^ although to date fewer such events have been ascertained. There are several reports that the phenotypic spectrum of 22q11.2dup is wide, including apparently unaffected transmitting parents.^11, 12^ As well as having a variety of physical manifestations, 22q11.2dup is reported to associate with ID and developmental delay in children^1, 12, 13^ and a wide range of psychiatric and behavioral abnormalities have been reported including attention-deficit hyperactivity disorder and autism, as well as other social and behavioral problems.^1, 12, 14^ The prevalence of the duplication in adults with psychiatric disorders has not been widely studied.

In the present study, we have established the rate of 22q11.2dup in a discovery sample of 6882 schizophrenia cases and 11 255 controls. The schizophrenia cases were genotyped on Illumina HumanOmniExpress-12v1 or HumanOmniExpressExome-8v1 arrays, and have been described elsewhere^15^ (Supplementary Material). The controls were obtained from four non-psychiatric data sets available through repositories, also genotyped on Illumina arrays (Supplementary Material). CNVs were detected using PennCNV.^16^ The probe set used for CNV calling was restricted to those common to all arrays used (520 766 probes). Full details of CNV calling and quality control are provided in the Supplementary Material. It should be noted that CNVs larger than 1 Mb are readily called with essentially perfect sensitivity by PennCNV on almost any SNP genotyping array. Significance of association was evaluated using a Fisher's exact test or a Cochran–Mantel–Haenszel test stratified by ethnicity and study. We also used RNAseq to determine the mRNA abundance in lymphoblastoid cell lines for 31 genes across the 22q11.2 CNV region (chr22:18 893 541–21 901 736, hg19) and genes 3 Mb either side, in 16 carriers of 22q11.21del, 6 carriers of 22q11.2dup and 821 individuals without a CNV at this locus.

In our discovery sample, we found no 22q11.2 duplications in schizophrenia cases (0%) but 10 (0.089%) in controls (Figure 1a, Table 1, Fisher's exact *P*=0.017). By way of contrast, we found reciprocal 22q11.2 deletions (a known strong risk factor) in 20 schizophrenia cases (0.29%) but in zero controls. No other CNV at any locus in the genome was found to be a putative protective factor at a nominal level of significance in this sample (data not shown).

Expression analysis of 22q11.2 deletion and duplication carriers indicated that the great majority of genes within the CNV region showed the expected increase or decrease in gene dosage, and that expression of genes flanking the CNV were not significantly affected by copy number change (Figure 1b). There have been relatively few systematic studies of gene expression in human 22q11.2 deletions and none of duplication carriers. Our data are highly congruent with a transcriptome-wide microarray study of RNA from untransformed peripheral blood mononuclear cells in showing significantly reduced expression of genes in the deleted region^17^ (further details in Supplementary Material).

In order to critically evaluate the reduced frequency of 22q11.2dup in cases in additional cohorts, we obtained data from the largest available CNV data sets known to us, in total comprising 14 256 additional cases and 14 612 additional controls. In this independent cohort, 22q11.2dups were also significantly rarer in cases than controls (0.021% vs 0.082%, Fisher's exact test *P*=0.020 and Table 1). A combined analysis of discovery and replication data found 22q11.2dup in 0.014% of cases and 0.085% of controls (Fisher's exact: *P*=0.00086, Cochran–Mantel–Haenszel: *P*=0.0019, OR=0.17, 95% confidence interval=0.05–0.56). The age of onset for the schizophrenia cases carrying the 22q11.2dup was 34, 28 and 43 years. Two of these cases have a history of seizures and none of them had any additional known pathogenic CNVs. Of the controls with a 22q11.2dup for which we have psychiatric data (*n*=6 from MGS sample), none had histories compatible with schizophrenia, schizoaffective disorder or major affective disorder. All graduated from high school and several had higher education degrees (confirming the notion that the duplication has incomplete penetrance for developmental delay).

Our study therefore identifies as the first putative protective mutation for schizophrenia duplications of the genomic segment that, when deleted, is the most potent genetic risk factor for the disorder. We can discount on several grounds the alternative explanation that those with 22q11.2dup have such a severe neurodevelopmental phenotype as to preclude a diagnosis of schizophrenia. First, as noted above, unaffected carriers are observed frequently (0.1% of controls across a large number of studies of psychiatric and non-psychiatric phenotypes). Second, the highly variable phenotype observed in 22q11.2dup carriers is often milder than that seen with many other schizophrenia-associated CNVs, as evidenced by its inheritance from an apparently unaffected parent in 69–74% of cases.^11, 12, 18, 19^ Most relevant to the present study, 22q11.2 deletions have consistently been reported to be associated with severe cognitive phenotypes such as autism and ID,^20^ but are seen at an appreciable rate in schizophrenia cohorts, including in the current samples (see above). If the duplication resulted in phenotypes inconsistent with inclusion in studies, we would expect greater depletion in controls, which are typically screened for health more intensively than cases, consistent with the finding of no 22q11 deletions among our controls. Finally, in the genome-wide analysis of our discovery sample, we also obtained evidence to support the association of deletions at 1q21.1, *NRXN1*, 3q29, 15q11.2, 15q13.3, 17q12 and 22q11.2 and duplications at 1q21.1, Williams–Beuren syndrome region, Prader–Willi/Angleman syndrome region, 16p13.11 and 16p11.2.^21^ These CNVs have all been associated with a similar range of neurodevelopmental phenotypes and our findings therefore argue strongly against the conclusion that our methods of ascertainment precluded the inclusion of cases carrying 22q11.2dup.

In all, 3 out of the 21 138 cases tested carried the 3 Mb 22q11.2 duplication, indicating that its putative protective effect is incomplete. However, our data suggest that its strength of effect may be strong, with an odds ratio less than 0.20 and an upper confidence limit of 0.56. The three case carriers had no additional pathogenic CNVs and we must assume that the development of schizophrenia resulted from other genetic or environmental factors. It was not possible to identify any particular characteristics of the cases carrying duplications but given their rarity power to do so was extremely limited.

To our knowledge, 22q11.2dup is the first putative protective mutation for schizophrenia that has been described in the literature. Our study suggests the existence of one or more dosage-sensitive gene in the duplication with the capacity to reduce risk of schizophrenia with implications here for further studies aimed at identifying targets for treating the disorder. This finding is of additional interest as this mutation does not appear to be similarly protective against the other neurodevelopmental phenotypes with which schizophrenia-associated CNVs are frequently associated.^1^ The present study in contrast provides a clear, opposite-direction dissociation between schizophrenia risk and both ID and autism spectrum disorder, both of which are more common in 22q11.2dup than in controls (Table 1). Neither the gene(s) nor the brain mechanisms by which 22q11.2del confers increased risk of neuropsychiatric and neurodevelopmental outcomes are known.^22^ However, that deletions are congruent in increasing risk of the disorders, whereas duplications act incongruently, simultaneously protecting against schizophrenia but predisposing to other neurodevelopmental disorders, suggests that at least some of the brain mechanisms are selective for schizophrenia. Moreover, that schizophrenia risk can potentially be reduced by a lesion that increases neurodevelopmental adversity (indexed by autism spectrum disorder and ID risk), suggests that the dosage-sensitive gene or genes might not just point the way to treatment, it may also hold clues to enhancing resilience among those who would generally be thought to be of elevated risk of the disorder.

Although large CNVs associate to risk of many disorders, it is generally unknown whether such effects arise from alterations of gene dosage or from other mechanisms. The lack (to date) of point mutations that phenocopy the neurodevelopmental and psychiatric effects of large deletions has invited alternative hypotheses, such as large-scale disruptions of chromatin or chromosomal pairing. Our finding that the reciprocal deletion and duplication of the same locus have potent risk and potential protective effects, respectively, for schizophrenia supports the hypothesis that one or more genes at 22q11.2 are dosage sensitive.^22^ The identification of the dosage-sensitive gene(s) at 22q11.2 and the implication of risk and protective mechanisms is therefore an important direction for research—particularly as pharmacological intervention might offer protection from schizophrenia. Our study was limited in its scope to implicate specific genes and possible mechanisms because CNVs at this locus affect multiple genes and the three duplications observed in cases of schizophrenia all involved the whole 3 Mb region. Further genetic studies on larger samples might inform this issue but it seems likely that a detailed understanding will only be provided by mechanistic studies in deletion and duplication carriers and in animal and cellular models.

## Figures and Tables

**Figure 1:**
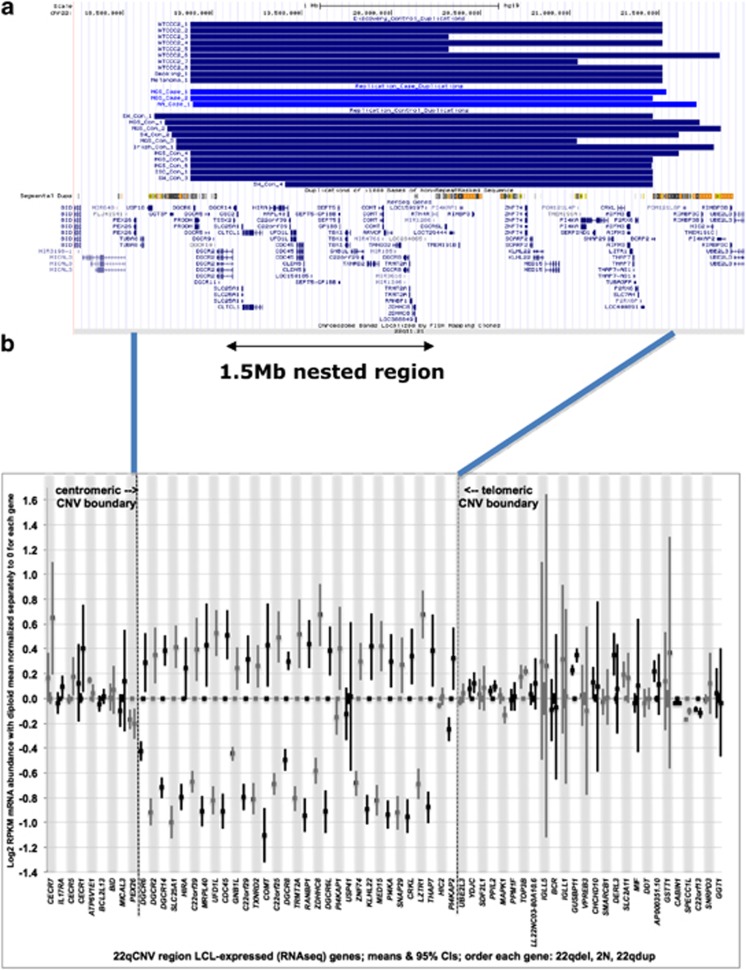
(**a**) Location of 22q11.2 duplications in discovery cases and replication cases and controls that cover the 1.5 Mb nested region. (**b**) Log_2_ RPKM mRNA abundance for genes in the 22q11 deletion and duplication carriers normalized to diploid subjects. Each gene (their positions depicted in alternating white or gray bars) has three measurements: deletion carriers, diploid subjects and duplication carriers. Genes are shown in map order, though their relative position is not drawn to scale. CNV, copy number variant.

**Table 1:** Frequencies of 22q11.2 duplications in cases and controls

*Study*	*Case 22q11.2dup frequency (*N *CNVs/*N *samples)*	*Control 22q11.2dup frequency (*N *CNVs/*N *samples)*	P *value (Fisher's exact test)*	*OR (95% CI)*
Discovery	0% (0/6 882)	0.089% (10/11 255)	0.017 (2-Tail)	
				
*Replication*				
MGS EA	0.090% (2/2 215)	0.16% (4/2 556)		
MGS AA	0% (0/977)	0.23% (2/881)		
ISC	0% (0/3 395)	0.031% (1/3 185)		
Irish/WTCCC2	0% (0/1 377)	0.10% (1/992)		
African American	0.061% (1/1 637)	0% (0/960)		
Swedish	0% (0/4 655)	0.066% (4/6 038)		
Total replication	0.021% (3/14 256)	0.082% (12/14 612)	0.020 (1-Tail)	
Total discovery+replication	0.014% (3/21 138)	0.085% (22/25 867)	0.00086 (2-Tail)	0.17 (0.050–0.56)
				
*Other disorders*				
ID/DD/CM	0.32% (50/15 767)	0.085% (23/27 133)	5.9 × 10^–8^ (2-Tail)	3.75 (2.29–6.15)
ASD	0.28% (12/4 315)	0.085% (23/27 133)	0.002 (2-Tail)	3.29 (1.63–6.61)

Abbreviations: AA, African American; ASD, autism spectrum disorder; CI, confidence interval; CM, congenital malformations; CNV, copy number variant; DD, developmental delay; EA, European American; ID, intellectual disability; ISC, International Schizophrenia Consortium; MGS, molecular genetics of schizophrenia; OR, odds ratio; WTCCC2, Wellcome Trust Case Control Consortium 2. The frequencies found in ID, DD, CM, ASD and their respective controls were taken from a recent review by Malhotra *et al.*^1^
