# Supplementary material for: Evidence that duplications of 22q11.2 protect against schizophrenia
Source: Mol Psychiatry. 2013 Nov 12;19(1):37–40. doi: 10.1038/mp.2013.156 (PMC3873028; doi:10.1038/mp.2013.156)
Supplement: Supplementary Table Legend [file mp2013156x2.doc]

Legend to Table S4

Mean log2 RPKM mRNA abundance and 95% confidence interval for each gene within and 3MB either side of the 22q11.21 consensus region, as calculated from 16 22q11.2 deletion carriers, 6 22q11.2 duplication carriers and 821 controls that are diploid for this locus. For further details see Supplementary Material.
