# Supplementary material for: Evidence that duplications of 22q11.2 protect against schizophrenia
Source: Mol Psychiatry. 2013 Nov 12;19(1):37–40. doi: 10.1038/mp.2013.156 (PMC3873028; doi:10.1038/mp.2013.156)
Supplement: Supplementary Materials [file mp2013156x3.doc]

**Supplementary Material**

**Duplications of 22q11.2 are protective for schizophrenia.**

1. Sample description
2. Discovery sample quality control
3. Control cross-dataset comparison
4. Log2 ratio and B-allele frequency traces for discovery duplication carriers
5. RNAseq methods
6. Acknowledgements

**1. Sample description**

**Discovery sample**

All 7 129 discovery cases came from samples we call the CLOZUK (n=6 558) and CardiffCOGS (n=571) series which have been described elsewhere^1^. Patients taking clozapine provide regular blood samples to allow early detection of adverse effects of that treatment. Through collaboration with Novartis, the manufacturer of a proprietary form of clozapine (Clozaril), we acquired blood from people with schizophrenia who were taking the drug via the central processing labs of a clozapine blood monitoring service. After the samples had been used to complete the necessary clinical tests, unused fractions were sent to Tepnel Life Sciences (Paisley, UK) for DNA extraction. Samples were anonymous, only basic demographic and diagnostic details being made available. Subjects (71% male) were UK residents, aged 18-90 with a recorded diagnosis of treatment resistant schizophrenia according to the clozapine registration forms completed by treating psychiatrists. In the UK, treatment resistant schizophrenia implies a lack of satisfactory clinical improvement to adequate trials of at least two other antipsychotics.

Approval by the local ethics committee was granted for the use of these samples in genetic association studies.

The CardiffCOGS is a sample of clinically diagnosed schizophrenic patients from the UK. Interview with the SCAN instrument^2^ and case note review was used to arrive at a best-estimate lifetime diagnosis according to DSM-IV criteria^3^.

All cases were genotyped on either HumanOmniExpress-12v1 or HumanOmniExpressExome-8v1 arrays at the Broad Institute, Cambridge, Massachusetts.

All controls for the discovery sample were downloaded with the relevant approvals for our study from the online repositories Database of Genotypes and Phenotypes (dbGaP) and the European Genome-Phenome Archive (EGA). The four non-psychiatric control datasets obtained, totalling 12 080 samples, are summarised in table S1. We purposefully selected datasets that were genotyped on high density Illumina arrays to ensure maximise probe overlap with the cases.

| **Dataset** | **Source (accession ID)** | **Array (N probes)** | **N Samples** |
| --- | --- | --- | --- |
| Schizophrenia Batch 1 | Broad Institute | HumanOmniExpress-12v1  (730 525) | 2 469 |
| Schizophrenia Batch 2 | Broad Institute | HumanOmniExpressExome-8v1  (951 117) | 3 621 |
| Schizophrenia Batch 3 | Broad Institute | HumanOmniExpressExome-8v1  (951 117) | 1 039 |
| The Genetic Architecture of **Smoking** and Smoking Cessation | dbGaP (phs000404.v1.p1) | Illumina HumanOmni2.5  (2 443 179) | 1 491 |
| High Density SNP Association Analysis of **Melanoma**: Case-Control and Outcomes Investigation | dbGaP (phs000187.v1.p1) | Illumina HumanOmni1_Quad_v1-0-B  (1 051 295) | 3 102 |
| Genetic Epidemiology of Refractive Error in the **KORA** Study | dbGaP (phs000303.v1.p1) | Illumina HumanOmni2.5  (2 443 179) | 1 869 |
| **WTCCC2** project samples from National Blood Donors (NBS) Cohort | EGA (EGAD00000000024) | Illumina 1.2M  (1 238 733) | 2 697 |
| **WTCCC2** project samples from 1958 British Birth Cohort | EGA (EGAD00000000022) | Illumina 1.2M  (1 238 733) | 2 921 |

**Table S1**. Summary of discovery cases and controls. Number of samples are those before quality control.

Principal component analysis (PCA) was performed to derive the ancestries of the discovery cases and controls by combining the data with Hapmap genotypes. Samples were stratified into those from a European (6 530 cases, 11 434 controls), African (263 cases, 478 controls) or ‘other’ (336 cases, 108 controls) origin.

**Replication samples**

**Molecular Genetics of Schizophrenia (MGS):** Details of the MGS cohort have been described elsewhere^4^. We processed the raw data and interrogated 22q11.2dups in 2 215 cases and 2 556 controls of European American ancestry and 977 cases and 881 controls of African American ancestry that passed our quality control. All schizophrenic patients met DSM-IV criteria^3^ for schizophrenia or schizoaffective disorder. The samples were genotyped at the Broad Institute, Cambridge, Massachusetts, using Affymetrix 6.0 genotyping arrays. CNVs were called using the Birdsuite algorithm^5^.

**International Schizophrenia Consortium (ISC):** Details of the ISC sample have been previously published^6^. The sample consists of six European populations genotyped at the Broad Institute, Cambridge, Massachusetts, using Affymetrix 6.0 or Affymetrix 5.0 genotyping arrays. We analysed CNVs in 3 395 cases and 3 185 controls.

**Irish/WTCCC2 sample:** Details of these samples have been published previously^7^. WTCCC2 samples that overlapped with our discovery sample were excluded by IBD analysis. Calls in the WTCCC2 schizophrenia sample were created using Birdseye from Birdsuite (version 1.5.5)^5^ for autosomes and we excluded calls where lengths were <100kb or >10Mb, or LOD score <10. We excluded CNVs with at least 50% overlap with other regional CNVs present in 1% or more of the samples. We excluded individuals with >30 CNV calls, or a total CNV length >10Mbp. Calls from plates containing fewer than 40 samples were also excluded.

**Swedish sample:** Subjects. See Ripke et al. for full description[^8^](#_ENREF_1). Briefly, all procedures were approved by ethical committees in Sweden and in the US, and all subjects provided written informed consent (or legal guardian consent and subject assent). Cases with schizophrenia were identified via the Swedish Hospital Discharge Register^9,10^ which captures all public and private inpatient hospitalizations. The register is complete from 1987 and augmented by psychiatric data from 1973-86. The register contains ICD discharge diagnoses [^11-13^](#_ENREF_4) made by attending physicians for each hospitalization. [^14-17^](#_ENREF_7) Case inclusion criteria: ≥2 hospitalizations with a discharge diagnosis of schizophrenia, both parents born in Scandinavia, and age ≥18 years. Case exclusion criteria: hospital register diagnosis of any medical or psychiatric disorder mitigating a confident diagnosis of schizophrenia as determined by expert review, and included removal of 3.4% of eligible cases due to the primacy of another psychiatric disorder (0.9%) or a general medical condition (0.3%) or uncertainties in the Hospital Discharge Register (e.g., contiguous admissions with brief total duration, 2.2%). The validity of this case definition of schizophrenia is strongly supported. Controls were selected at random from Swedish population registers with the goal of obtaining an appropriate control group and avoiding “super-normal” controls[^18^](#_ENREF_11). Control inclusion criteria: never hospitalized for schizophrenia or bipolar disorder (given evidence of genetic overlap with schizophrenia), [^19-21^](#_ENREF_12) both parents born in Scandinavia, and age ≥18 years. The sample was approximately representative of the Swedish populace in regard to county of birth.

Genotyping, quality control, and imputation. DNA was extracted from peripheral blood samples at the Karolinska Institutet Biobank. Samples were genotyped in six batches at the Broad Institute using Affymetrix 5.0 (3.9%), Affymetrix 6.0 (38.6%), and Illumina OmniExpress (57.4%) chips according to the manufacturers’ protocols. Genotype calling, quality control, and imputation were done in four sets corresponding to data from Affymetrix 5.0 (Sw1), Affymetrix 6.0 (Sw2-4), and the OmniExpress batches (Sw5, Sw6). Genotypes were called using Birdsuite (Affymetrix) or BeadStudio (Illumina). The quality control parameters applied were: SNP missingness < 0.05 (before sample removal); subject missingness < 0.02; autosomal heterozygosity deviation; SNP missingness < 0.02 (after sample removal); difference in SNP missingness between cases and controls < 0.02; and deviation from Hardy-Weinberg equilibrium (*P* < 10^−6^ in controls or *P* < 10^−10^ in cases).

The Birdseye tool in Birdsuite^5^ was applied to intensity data from SNP and CNV probes. The Birdseye algorithm uses a hidden Markov model (HMM) approach to find regions of variable copy number in a sample. Model priors were generated for each genotyping platform. All genomic positions were mapped to the hg19 coordinates.

A multi-step quality control (QC) procedure was implemented in order to assemble a high-quality rare CNV callset. Samples were excluded if they failed SNP QC or if they had > 40 CNV calls or > 10Mb of CNVs^6^. CNVs were excluded if they were of low confidence (LOD <10, size < 20kb, or spanning < 10 probes) or if they overlapped large genomic gaps (≥1kb overlap). Any CNVs that appeared to be artificially split by the HMM were annealed. Next, we imposed a 1% frequency threshold by removing any CNV with > 50% of its length spanning a region with CNVs from >1% of total samples as implemented in PLINK^22^. Finally, we extracted large CNVs that are ≥100kb in length resulting in a total of 10 161 CNV segments in 4 655 cases and 6 038 controls.

**African American sample:** The Genomic Psychiatry Cohort (GPC) is a clinical cohort of patients enrolled at sites across the United States, in a collaboration directed by Drs. Michele and Carlos Pato at USC. Psychiatric diagnoses were made through personal interviews and review of the medical records. Interviews were performed by trained clinicians using a structured psychiatric interview instrument, the Diagnostic Interview for Psychosis and Affective Disorder (DI-PAD), to asses participants.  The DI-PAD is based on the Diagnostic Interview for Genetic Studies (DIGS)^23^ and includes 90 phenomenological symptom items that are used to arrive at final diagnoses under various diagnostic criteria. Clinicians reviewed diagnoses that were based on DSM-IV^3^. Cases were included in the current study if they met criteria for schizophrenia or schizoaffective disorder. Individuals without a personal or family history of psychosis or mania were eligible to participate as controls.  In the current study, we genotyped samples from the GPC cohort members with self-reported African American ancestry.

CNVs were called on all samples using PennCNV and NCBI37/hg19 coordinates. The following samples were removed: duplicate individuals, first degree relatives (if discordant phenotypes, always the control was removed), individuals with more than 2% missing genotypes, individuals with more than 60% European ancestry, individuals with more than 10Mbp of the genome estimated as CNV.

1. **Discovery sample quality control**

Raw intensity data from each case/control dataset were independently processed and analysed to account for potential batch effects. Log2 ratios and B-allele frequencies were generated using Illumina Genome Studio software (v2011.1). CNVs were called using the PennCNV calling algorithm, following the standard protocol and adjusting for GC content. The 520 766 probes common to all discovery arrays were used for CNV calling. Samples were excluded if for any one of the following QC metrics they represented an outlier in their source dataset: Log2 ratio standard deviation, B-allele frequency drift, wave factor and total number of CNVs called per person. Table S2 shows the number of samples that failed QC from each discovery dataset. As some of these data were already filtered for quality before they were downloaded, the proportions of failed samples across the datasets are not comparable.

| **Sample** | **Total Excluded** | **Total Retained** | **Ethnicity** | | |
| --- | --- | --- | --- | --- | --- |
|  |  |  | **European (retained)** | **African (retained)** | **Other (retained)** |
| SCZ | 247 | 6882 | 6530 (6307) | 263 (251) | 336 (324) |
| Smoking | 3 | 1488 | 939 (938) | 478 (478) | 74 (72) |
| Melanoma | 131 | 2971 | 3086 (2955) | 0 | 16 (16) |
| NBS_WTCCC1 | 140 | 1165 | 1297 (1159) | 0 | 8 (6) |
| 58_WTCCC1 | 152 | 1248 | 1398 (1247) | 0 | 2 (1) |
| NBS_WTCCC2 | 182 | 1210 | 1386 (1204) | 0 | 6 (6) |
| 58_WTCCC2 | 205 | 1316 | 1519 (1315) | 0 | 2 (1) |
| KORA | 12 | 1857 | 1869 (1857) | 0 | 0 |
| Total | 1072 | 18137 |  |  |  |

**Table S2**. Number of case and control discovery samples before and after QC and their ethnicities.

Following the exclusion of poorly performing samples, we performed quality control on the called CNVs. Firstly, CNVs in the same individual were joined if the distance separating them was less than 50% of their combined length. All CNVs were then excluded if they were covered by less than 10 probes, were less than 15kb in length, overlapped with low copy repeats by more than 50% of their length, or had a probe density (calculated by dividing the size of the CNV by the number of probes covering it) greater than 20k.

1. **Control cross-dataset comparison**

As the discovery and replication samples consist of several different datasets, we tested whether an unknown ascertainment bias could have potentially caused the observed rates of 22q11.2 duplications by comparing the rates found across each control dataset with a 2-sided Fisher’s Exact test (Table S3). Despite being ascertained at different times and locations, no two control datasets were found to be statistically different to each other.

| 2-sided Fisher’s Exact P-value | | | | | | | | | | |
| --- | --- | --- | --- | --- | --- | --- | --- | --- | --- | --- |
|  | WTCCC2 | Melanoma | Smoking | Kora | MGS EA | MGS AA | ISC | Irish | African | Swedish |
| WTCCC2 |  | 0.17 | 0.69 | 0.12 | 1 | 0.65 | 0.1 | 1 | 0.37 | 0.15 |
| Melanoma | 0.17 |  | 1 | 1 | 0.19 | 0.13 | 1 | 0.44 | 1 | 1 |
| Smoking | 0.69 | 1 |  | 0.44 | 0.66 | 0.56 | 0.54 | 1 | 1 | 1 |
| Kora | 0.12 | 1 | 0.44 |  | 0.14 | 0.1 | 1 | 0.35 | 1 | 0.58 |
| MGS EA | 1 | 0.19 | 0.66 | 0.14 |  | 0.65 | 0.18 | 1 | 0.58 | 0.25 |
| MGS AA | 0.65 | 0.13 | 0.56 | 0.1 | 0.65 |  | 0.12 | 0.6 | 0.23 | 0.17 |
| ISC | 0.1 | 1 | 0.54 | 1 | 0.18 | 0.12 |  | 0.42 | 1 | 0.67 |
| Irish | 1 | 0.44 | 1 | 0.35 | 1 | 0.6 | 0.42 |  | 1 | 0.53 |
| African | 0.37 | 1 | 1 | 1 | 0.58 | 0.23 | 1 | 1 |  | 1 |
| Swedish | 0.15 | 1 | 1 | 0.58 | 0.25 | 0.17 | 0.67 | 0.53 | 1 |  |

**Table S3.** Comparison of 22q11.2 duplication rate in all control datasets. 2-sided Fisher’s Exact test p values are shown.

1. **Log2 ratio and B-allele frequency traces for discovery duplication carriers**

In our analysis, the nested 1.5Mb 22q11.2 region is covered by ~368 probes and the larger 3 Mb region by ~539 probes. Given the size and probe coverage of these CNVs, we would expect a very high specificity and sensitivity for 22q11.2 CNV calling. We manually checked the log2 ratio and B-allele frequency traces which confirmed all duplications (Figure S1).

**A**

**1.5Mb nested region**

**B**

**1.5Mb nested region**

**C**

**1.5Mb nested region**

**Figure S1**. Log2 ratios and B-allele frequencies for discovery sample 22q11.2 duplication carriers. **A**, WTCCC2 data, **B** Melanoma data, **C** Smoking data.

1. **RNAseq Methods**

We sequenced mRNA extracted from 843 lymphoblastoid cell lines (LCLs) of subjects from the Molecular Genetics of Schizophrenia (MGS) collection^4^, including 22q11.21 deletion-carrying (N=16) and duplication-carrying (N=6) subjects^4^, at a depth of 60 million reads/sample for CNV carriers and 10 million reads/sample for the non-CNV-carrying subjects. We aligned the 50bp single reads to the human reference gene map Gencode v14 using the mapping tool Tophat v2.0.5, allowing for 2 mismatches. We calculated gene expression levels as RPKM values from raw reads counts generated from HTseq-count script^23^. We defined the consensus 22q11.21 CNV region as the minimum starting position and maximum ending position from any MGS 22q11.21 CNV carrier^4^. We calculated for each group (subjects with 22q11.21 deletions, duplications, or normal 2N) the mean log_2_ RPKM and its 95% confidence interval for each gene within 3MB of the start and 3MB of the end of the 22q11.21 consensus region, and within the CNV boundaries as well (Table S4). We present data here without regressing out covariates (e.g., sex, age, ancestry, LCL characteristics) for the gene expression levels, but note that the plots are very similar when regressing out such covariates (data not shown). For ease of viewing, we individually (by gene) zeroed the log_2_ RPKM values by subtracting each gene’s mean log_2_ RPKM values for the non-CNV-carriers from that gene’s log_2_ RPKM values (figure 1b main text).

**Previous reports of human 22q11.2 CNV gene expression**

To our knowledge, few expression studies of human 22q11.2 CNV deletion carriers have been reported.  The largest study in terms of sample size used qPCR to assay RNA from blood on 38 carriers of the 22q11.2 CNV deletion versus 16 diploid controls, examining DGCR6 and DGCR6L^24^, and found no differential expression. In contrast, we found each gene to be expressed at significantly lower levels in 16 carriers of the 22q11.2 CNV deletion compared with 821 diploid controls our study on LCLs.

In a transcriptome-wide study performed on RNA from untransformed peripheral blood mononuclear cells (PBMCs) assayed by microarray on seven 22q11.2 CNV deletion carriers versus seven diploid controls, numerous 22q11.2 CNV region genes were found to be expressed at lower levels in the deletion carriers^25^: (1) By the significance analysis of microarrays (SAM) method, nine genes in the CNV region were differentially expressed. (2) By the locally adaptive statistical (LAP) procedure, 25 genes (when using the same CNV boundaries as us) in the CNV region were differentially expressed.  Our RNAseq on LCLs findings were highly congruent with the microarray study on PBMCs^25^: we found 9/9 of the SAM identified genes and 22/25 of the LAP identified genes to be expressed at significantly lower levels.  We found no transcriptomic studies on other human tissues (e.g., postmortem brain, LCLs) in 22q11.2 CNV deletion carriers, and no expression studies on 22q11.2 CNV duplication carriers at all, in the literature.

1. **Acknowledgements**

**Members of Wellcome Trust Case Control Consortium 2**

Management Committee

Peter Donnelly (Chair)^1,2^, Ines Barroso (Deputy Chair)^3^, Jenefer M Blackwell^4, 5^,  Elvira Bramon^6^ , Matthew A Brown^7^ , Juan P Casas^8^ , Aiden Corvin^9^, Panos Deloukas^3^, Audrey Duncanson^10^, Janusz Jankowski^11^, Hugh S Markus^12^, Christopher G Mathew^13^, Colin NA Palmer^14^, Robert Plomin^15^, Anna Rautanen^1^, Stephen J Sawcer^16^, Richard C Trembath^13^, Ananth C Viswanathan^17^, Nicholas W Wood^18^

Data and Analysis Group

Chris C A Spencer^1^, Gavin Band^1^, Céline Bellenguez^1^, Colin Freeman^1^, Garrett Hellenthal^1^, Eleni Giannoulatou^1^, Matti Pirinen^1^, Richard Pearson^1^, Amy Strange^1^, Zhan Su^1^, Damjan Vukcevic^1^, Peter Donnelly^1,2^

DNA, Genotyping, Data QC and Informatics Group

Cordelia Langford^3^, Sarah E Hunt^3^, Sarah Edkins^3^, Rhian Gwilliam^3^, Hannah Blackburn^3^, Suzannah J Bumpstead^3^, Serge Dronov^3^, Matthew Gillman^3^, Emma Gray^3^, Naomi Hammond^3^, Alagurevathi Jayakumar^3^, Owen T McCann^3^, Jennifer Liddle^3^, Simon C Potter^3^, Radhi Ravindrarajah^3^, Michelle Ricketts^3^, Matthew Waller^3^, Paul Weston^3^, Sara Widaa^3^, Pamela Whittaker^3^, Ines Barroso^3^, Panos Deloukas^3^**.**

Publications Committee

Christopher G Mathew (Chair)^13^, Jenefer M Blackwell^4,5^, Matthew A Brown^7^, Aiden Corvin^9^, Chris C A Spencer^1^

1 Wellcome Trust Centre for Human Genetics, University of Oxford, Roosevelt Drive, Oxford OX3 7BN, UK; 2 Dept Statistics, University of Oxford, Oxford OX1 3TG, UK; 3 Wellcome Trust Sanger Institute, Wellcome Trust Genome Campus, Hinxton, Cambridge CB10 1SA, UK; 4 Telethon Institute for Child Health Research, Centre for Child Health Research, University of Western Australia, 100 Roberts Road, Subiaco, Western Australia 6008; 5 Cambridge Institute for Medical Research, University of Cambridge School of Clinical Medicine, Cambridge CB2 0XY, UK; 6 Department of Psychosis Studies, NIHR Biomedical Research Centre for Mental Health at the Institute of Psychiatry, King’s College London and The South London and Maudsley NHS Foundation Trust, Denmark Hill, London SE5 8AF, UK; 7 University of Queensland Diamantina Institute, Brisbane, Queensland, Australia; 8 Dept Epidemiology and Population Health, London School of Hygiene and Tropical Medicine, London WC1E 7HT and Dept Epidemiology and Public Health, University College London WC1E6BT, UK; 9 Neuropsychiatric Genetics Research Group, Institute of MolecularMedicine, Trinity College Dublin, Dublin 2, Eire; 10 Molecular and Physiological Sciences, The Wellcome Trust, London NW1 2BE; 11 Department of Oncology, Old Road Campus, University of Oxford, Oxford OX3 7DQ, UK , Digestive Diseases Centre, Leicester Royal Infirmary, Leicester LE7 7HH, UK and Centre for Digestive Diseases, Queen Mary University of London, London E1 2AD, UK; 12 Clinical Neurosciences, St George's University of London, London SW17 0RE; 13 King’s College London Dept Medical and Molecular Genetics, King’s Health Partners, Guy’s Hospital, London SE1 9RT, UK; 14 Biomedical Research Centre, Ninewells Hospital and Medical School, Dundee DD1 9SY, UK; 15 King’s College London Social, Genetic and Developmental Psychiatry Centre, Institute of Psychiatry, Denmark Hill, London SE5 8AF, UK; 16 University of Cambridge Dept Clinical Neurosciences, Addenbrooke’s Hospital, Cambridge CB2 0QQ, UK; 17 NIHR Biomedical Research Centre for Ophthalmology, Moorfields Eye Hospital NHS Foundation Trust and UCL Institute of Ophthalmology, London EC1V 2PD, UK; 18 Dept Molecular Neuroscience, Institute of Neurology, Queen Square, London WC1N 3BG, UK.

References

1 Hamshere, ML, Walters, JTR, Smith, R, Richards, AL, Green, E, Grozeva, D, Jones, I, Forty, L, Jones, L, Gordon-Smith, K, Riley, B, O'Neill, T, Kendler, KS, Sklar, P, Purcell, S, Kranz, J, Morris, D, Gill, M, Holmans, P, Craddock, N, Corvin, A, Owen, MJ & O'Donovan, MC. Genome-wide significant associations in schizophrenia to ITIH3/4, CACNA1C and SDCCAG8, and extensive replication of associations reported by the Schizophrenia PGC. *Mol Psychiatry* 2012; **18**: 708-712.

2 Wing, JK, Babor, T, Brugha, T, Burke, J, Cooper, JE, Giel, R, Jablenski, A, Regier, D & Sartorius, N. SCAN: Schedules for Clinical Assessment in Neuropsychiatry. *Arch. Gen. Psychiatry.* 1990; **47**: 589-593.

3 American Psychiatric Association. *Diagnostic and Statistical Manual of Mental Disorders*. Fourth edn, (American Psychiatric Press, 1994).

4 Levinson, DF, Duan, J, Oh, S, Wang, K, Sanders, AR, Shi, J, Zhang, N, Mowry, BJ, Olincy, A, Amin, F, Cloninger, CR, Silverman, JM, Buccola, NG, Byerley, WF, Black, DW, Kendler, KS, Freedman, R, Dudbridge, F, Pe'er, I, Hakonarson, H, Bergen, SE, Fanous, AH, Holmans, PA & Gejman, PV. Copy number variants in schizophrenia: confirmation of five previous findings and new evidence for 3q29 microdeletions and VIPR2 duplications. *Am J Psychiatry* 2011; **168**: 302-316.

5 Korn, JM, Kuruvilla, FG, McCarroll, SA, Wysoker, A, Nemesh, J, Cawley, S, Hubbell, E, Veitch, J, Collins, PJ, Darvishi, K, Lee, C, Nizzari, MM, Gabriel, SB, Purcell, S, Daly, MJ & Altshuler, D. Integrated genotype calling and association analysis of SNPs, common copy number polymorphisms and rare CNVs. *Nat Genet* 2008; **40**: 1253-1260.

6 International Schizophrenia Consortium (ISC). Rare chromosomal deletions and duplications increase risk of schizophrenia. *Nature* 2008; **455**: 237-241.

7 Irish Schizophrenia Genomics Consortium and the Wellcome Trust Case Control Consortium 2. Genome-Wide Association Study Implicates HLA-C*01:02 as a Risk Factor at the Major Histocompatibility Complex Locus in Schizophrenia. *Biol. Psychiatry* 2012; **72**: 620-628.

8 Ripke, S, O'Dushlaine, C, Chambert, K, Moran, JL, Kähler, A, Akterin, S, Bergen, S, Collins, AL, Crowley, J, Fromer, M, Kim, Y, Lee, SH, Magnusson, PK, Sanchez, N, Stahl, E, Williams, S, Wray, N, Xia, K, Bettella, F, Børglum , AD, Cormican, P, Craddock, N, de Leeuw, C, Durmishi, N, Gill, M, Golimbet, V, Hamshere, ML, Holmans, P, Hougaard, DM, Kendler, KS, Lin, K, Morris, DW, Mors, O, Mortensen, PB, Neale, B, O'Neill, FA, Owen, MJ, Pejovic Milovancevic, M, Posthuma, D, Powell, J, Richards, AL, Riley, BP, Ruderfer, D, Rujescu, D, Sigurdsson, E, Silagadze, T, Smit, AB, Stefansson, H, Steinberg, S, Suvisaari, J, Tosato, S, Walters, JT, Verhage, M, Multicenter Genetic Studies of Schizophrenia Consortium, Psychosis Endophenotypes Consortium, Wellcome Trust Case-Control Consortium2, Bramon, E, Corvin, AP, O'Donovan, MC, Stefansson, K, Scolnick, E, Purcell, S, McCarroll, S, Sklar, P, Hultman, C & Sullivan, PF. Genome-wide association of schizophrenia in Sweden. Submitted;

9 Kristjansson, E, Allebeck, P & Wistedt, B. Validity of the diagnosis of schizophrenia in a psychiatric inpatient register. *Nordisk Psykiatrik Tidsskrift* 1987; **41**: 229-234.

10 Dalman, C, Broms, J, Cullberg, J & Allebeck, P. Young cases of schizophrenia identified in a national inpatient register--are the diagnoses valid? *Social Psychiatry and Psychiatric Epidemiology* 2002; **37**: 527-531.

11 World Health Organization. *International Classification of Diseases*. 8th revised edn, (World Health Organization, 1967).

12 World Health Organization. *International Classification of Diseases*. 9th revised edn, (World Health Organization, 1978).

13 World Health Organization. *International Classification of Diseases*. 10th revised edn, (World Health Organization, 1992).

14 Hultman, CM, Sparen, P, Takei, N, Murray, RM & Cnattingius, S. Prenatal and perinatal risk factors for schizophrenia, affective psychosis, and reactive psychosis of early onset: case-control study. *Bmj* 1999; **318**: 421-426.

15 Zammit, S, Allebeck, P, Dalman, C, Lundberg, I, Hemmingsson, T & Lewis, G. Investigating the association between cigarette smoking and schizophrenia in a cohort study. *Am J Psychiatry* 2003; **160**: 2216-2221.

16 Andersson, RE, Olaison, G, Tysk, C & Ekbom, A. Appendectomy and protection against ulcerative colitis. *N Engl J Med* 2001; **344**: 808-814.

17 Hansson, LE, Nyren, O, Hsing, AW, Bergstrom, R, Josefsson, S, Chow, WH, Fraumeni, JF, Jr. & Adami, HO. The risk of stomach cancer in patients with gastric or duodenal ulcer disease. *N Engl J Med* 1996; **335**: 242-249.

18 Schwartz, S & Susser, E. Genome-wide association studies: does only size matter? *Am J Psychiatry* 2010; **167**: 741-744.

19 Craddock, N & Owen, MJ. The Kraepelinian dichotomy - going, going... but still not gone. *Br J Psychiatry* 2010; **196**: 92-95.

20 International Schizophrenia Consortium. Common polygenic variation contributes to risk of schizophrenia and bipolar disorder. *Nature* 2009; **460**: 748-752.

21 Lichtenstein, P, Yip, B, Bjork, C, Pawitan, Y, Cannon, TD, Sullivan, PF & Hultman, CM. Common genetic influences for schizophrenia and bipolar disorder: A population-based study of 2 million nuclear families. *Lancet* 2009; **373**: 234-239.

22 Purcell, S, Neale, B, Todd-Brown, K, Thomas, L, Ferreira, MAR, Bender, D, Maller, J, Sklar, P, de Bakker, PIW, Daly, MJ & Sham, PC. PLINK: A Tool Set for Whole-Genome Association and Population-Based Linkage Analyses. *Am J Hum Genet* 2007; **81**: 559-575.

23 Anders, S & Huber, W. Differential expression analysis for sequence count data. *Genome Biol.* 2010; **11**: R106.

24 Chakraborty, D, Bernal, AJ, Schoch, K, Howard, TD, Ip, EH, Hooper, SR, Keshavan, MS, Jirtle, RL & Shashi, V. Dysregulation of DGCR6 and DGCR6L: psychopathological outcomes in chromosome 22q11.2 deletion syndrome. *Transl Psychiatry* 2012; **2**: e105.

25 van Beveren, NJM, Krab, LC, Swagemakers, S, Buitendijk, G, Boot, E, van der Spek, P, Elgersma, Y & van Amelsvoort, TAMJ. Functional Gene-Expression Analysis Shows Involvement of Schizophrenia-Relevant Pathways in Patients with 22q11 Deletion Syndrome. *PLoS ONE* 2012; **7**: e33473.

Legend to Table S4

Mean log2 RPKM mRNA abundance and 95% confidence interval for each gene within and 3MB either side of the 22q11.21 consensus region, as calculated from 16 22q11.2 deletion carriers, 6 22q11.2 duplication carriers and 821 controls that are diploid for this locus. For further details see Supplementary Material.
